# Functional connectivity during cognitive control in children with autism spectrum disorder: an independent component analysis

**DOI:** 10.1007/s00702-014-1237-8

**Published:** 2014-05-21

**Authors:** S. Ambrosino, D. J. Bos, T. R. van Raalten, N. A. Kobussen, J. van Belle, B. Oranje, S. Durston

**Affiliations:** NICHE Lab, Department of Psychiatry, Brain Center Rudolf Magnus, University Medical Center Utrecht, HP A.01.126, Heidelberglaan 100, 3584 CX Utrecht, The Netherlands

**Keywords:** ASD, Functional connectivity, ICA, Cognitive control, Rigid behavior

## Abstract

Restrictive and repetitive behavior in autism may be related to deficits in cognitive control. Here, we aimed to assess functional connectivity during a cognitive control task and compare brain network activity and connectivity in children with autism spectrum disorders (ASD) and typically developing children using a multivariate data-driven approach. 19 high-functioning boys with ASD and 19 age-matched typically developing boys were included in this study. Functional magnetic resonance imaging was performed at 3T during the performance of a cognitive control task (go/no-go paradigm). Functional networks were identified using independent component analysis. Network activity and connectivity was compared between groups and correlated with clinical measures of rigid behavior using multivariate analysis of covariance. We found no differences between the groups in task performance or in network activity. Power analysis indicated that, if this were a real difference, it would require nearly 800 subjects to show group differences in network activity using this paradigm. Neither were there correlations between network activity and rigid behavior. Our data do not provide support for the presence of deficits in cognitive control in children with ASD, or the functional networks supporting this ability.

**Electronic supplementary material:**

The online version of this article (doi:10.1007/s00702-014-1237-8) contains supplementary material, which is available to authorized users.

## Introduction

Autism spectrum disorders (ASD) are characterized by three defining symptom clusters: impairments in social interaction, communication difficulties, and restrictive, repetitive and stereotyped patterns of behaviors (American Psychiatric Association 2000). It has been suggested that different aetiological processes contribute to these disorders, and one useful way to study more homogeneous subgroups may be to focus on core areas of symptoms (Langen et al. 2011a, b). The cluster of rigid behavior may in part reflect problems in cognitive control (Hill 2004; Solomon et al. 2008). Cognitive control comprises a wide range of abilities that help maintain an appropriate cognitive set in working memory to achieve a later goal, such as planning, mental flexibility, sustained attention, interference inhibition, response suppression (or inhibitory control), outcome monitoring and the ability to deal with novelty (Chan et al. 2008). Some behavioral manifestations of rigidity in ASD seem particularly related to motor-response inhibition (Mosconi et al. 2009). Rigid behavior could then reflect the inability to inhibit pre-potent or ongoing motor behaviors when they are no longer appropriate, resulting in an inability to favor the expression of other, more adaptive responses.

Functional magnetic resonance imaging (fMRI) studies have shown activation of a network of brain regions during the execution of cognitive control tasks, including prefrontal cortex, anterior cingulate cortex, striatum and posterior parietal cortex. This network of functionally connected regions has been termed the cognitive control network (Durston and Casey 2006; Cole and Schneider 2007). However, effective cognitive control is also related to the concurrent modulation of other networks, i.e. coactivation of the salience network (Menon and Uddin 2010) and deactivation of the default mode network (Buckner et al. 2008; Raichle et al. 2001) during cognitive control tasks.

Several fMRI studies of cognitive control have reported atypical activation in task-related areas in individuals with ASD compared to controls, particularly in the frontostriatal circuitry (for review, see Dichter 2012). Only few studies of functional connectivity during cognitive control have been conducted and most of them report reduced connectivity in the cognitive control network and related brain regions in ASD (Just et al. 2007; Kana et al. 2007; Solomon et al. 2009; Agam et al. 2010). This could be taken to suggest immature functional integration or segregation of networks in ASD. Furthermore, it suggests that symptoms of ASD, such as rigid behavior, may be related to underconnectivity of functional networks rather than to changes in the discrete regions of the cognitive control network. This would support the developmental disconnection hypothesis as an explanatory model for deficits in executive functioning in ASD (Geschwind and Levitt 2007).

In the current study we aimed to investigate connectivity both within and between functional networks involved in cognitive control in a group of high-functioning boys with ASD and age-matched typically developing boys. We used independent component analysis (ICA) to identify cognitive control networks and investigate their activity and connectivity. ICA is a data-driven method that decomposes fMRI data into spatially independent, but temporally coherent networks (Calhoun et al. 2001, 2002a, 2009; Calhoun and Adali 2006). Decomposition into networks in this manner greatly reduces the number of comparisons made compared to standard GLM analyses. As such, ICA is more sensitive to between-group differences than a traditional GLM analysis (Congdon et al. 2010; McKeown and Sejnowski 1998). In addition, ICA allows one specific voxel to contribute to more than one temporally coherent network, and as such it may be involved in more than one pattern of response. Therefore, ICA may even detect differences that are obscured in traditional GLM analyses (Beldzik et al. 2013; Xu et al. 2013a). Based on the developmental disconnectivity hypothesis of ASD, we hypothesized: (1) reduced connectivity between cognitive control and other task-related networks in ASD; and (2) that reduced connectivity of the cognitive control network would be related to severity of rigid behavior in ASD.

## Methods

### Participants and clinical data

A total of 38 boys, 19 with a diagnosis of ASD (aged 9–14 years) and 19 age-matched typically developing boys, were included in the study. In addition to age, participants were matched at the group level for hand preference and IQ. The study and its procedures were approved by the Institutional Review Board of the University Medical Centre Utrecht, The Netherlands. Written informed consent was obtained from the parents of all subjects after full disclosure of the study purpose and procedure. Children provided written and/or verbal informed assent.

For participants with ASD, a qualified researcher from the lab confirmed the clinical diagnosis by means of the Autism Diagnostic Interview-Revised (ADI-R) (Lord et al. 1994). The Diagnostic Interview Schedule for Children (DISC, version 2.3 or IV), parent version (Shaffer et al. 2000), was administered to parents of the typically developing children in order to confirm the absence of any psychiatric diagnosis in the participant. In addition, controls were excluded in case of first-degree relatives with a history of psychiatric problems. In both groups, additional exclusion criteria were IQ below 70, any major physical or neurological illnesses, or the presence of metal in the body that precluded the MRI session.

The Repetitive Behavior Scale Revised (RBS-R) was administered to provide a quantitative measure of the full spectrum of repetitive behaviors in ASD participants (Bodfish et al. 1999, 2000); the scale includes measures of stereotyped, self-injurious, compulsive, and ritualistic behavior, insistence on sameness and restricted interests. Full scale IQ was assessed with the Wechsler intelligence scale for children WISC-III (Wechsler 2005). Table 1 lists the demographic and clinical characteristics of the sample; the appropriate parametric, non-parametric, Chi-squared or Fisher exact tests were performed to test for between-group differences on these variables.

**Table 1:** Demographics and clinical characteristics

	ASD (*N* = 19)	Controls (*N* = 19)	Group differences (*p* values)
Age			
*M* (SD)	11.5 (1.2)	11.1 (1.6)	.367
Range	9.0–12.8	9.1–14.2	
Total IQ^a^			
*M* (SD)	112.2 (15.3)	120.2 (15.8)	.134
Range	80–150	88–152	
Handedness			
N Right/ambidextrous/left	19/0/0	17/2/0	.486
SES^b^			
Education father (years) *M* (SD)	14.5 (0.5)	13.9 (2.6)	.50
ADI-R social			
*M* (SD)	20.6 (4.3)		
ADI-R communication			
*M* (SD)	15.2 (4.3)		
ADI-R repetitive			
*M* (SD)	6.0 (2.6)		
Total RBS-R^c^			
*M* (SD)	24.9 (15.5)		
Medication			
*N* Medicated/unmedicated	7^d^/12	0/19	.008

*ASD* autism spectrum disorder, *N* number, *M* mean, *SD* standard deviation, *IQ* intelligence quotient, *SES* socio-economic status, *ADI-R* autism diagnostic interview revised, *RBS-R* repetitive behavior scale revised

^a^Unavailable for two subjects with ASD; ^b^ unavailable for ten controls and thirteen subjects with ASD; ^c^ unavailable for one subject with ASD; ^d^five children on methylphenidate, three children on risperidone

Seven children with ASD were on psychoactive medication at the time of study. The five children with ASD that were on methylphenidate were instructed not to take their medication for at least 24 h prior to the scanning session. As this is not possible for risperidone due to a longer washout period, the use of risperidone was permitted for three subjects with ASD. All other participants were medication-naïve. Prior to the MRI scanning, children under 13 years of age were acclimated to the MRI procedure in a practice session using a mock scanner as described by Durston et al. (2009); subjects aged 13 years or over were also offered the opportunity to do a practice session. Participants were scanned only in case of a successful practice session.

### Task design

All subjects participated in an fMRI-session, during which they performed a go/no-go task, as described previously (Durston et al. 2002a, b, 2003, 2006), in short: participants were instructed to focus on a centrally presented fixation point, and to respond as fast as possible to visually presented go stimuli with a button press, and to withhold responding when a rare non-target was presented (no-go). In order to make the task interesting for children, Pokémon characters were used as stimuli. The task consisted of four sessions of equal length (3 min 56 s). Each run contained a total of 57 trials, with 25 % no-go trials. No-go trials were preceded by 1, 3 or 5 go trials in pseudo-randomized order. Each stimulus was displayed for 500 ms, followed by an interval of 3,500 ms. Stimuli were projected using a through-projection screen and slide projector. Behavioral responses were collected using a magnet-compatible air pressure button device.

### Statistical analysis of task performance

SPSS Statistics version 20.0.0 for Mac OS X (SPSS Inc., Chicago, IL) was used for the analyses of the behavioral measures from the task. Accuracy on go-trials and accuracy on no-go trials (mean accuracy and following 1, 3 or 5 preceding go-trials) were calculated. Mean reaction time on successful go-trials was measured.

Developmental effects were investigated by calculating Pearson’s correlations (*r*) between age and behavioral measures. In the ASD group only, *r* coefficients were calculated to investigate the correlation between symptoms of rigidity (RBS-R total score) and performance parameters. Group differences in task performance were investigated using a univariate general linear model, with age at scan and age-by-diagnosis interaction entered as covariates. An uncorrected alpha level of 0.05 was used for these analyses.

### fMRI acquisition

Data were acquired using a 3.0 T Philips Allegra MRI scanner (Philips Medical Systems, Best, The Netherlands). Task-related functional images were collected in 4 runs of 119 frames with a 2D-EPI SENSE sequence (TR/TE 2,000/35 ms, flip angle 70°, matrix 68 × 66, FOV 24 cm, voxel size 3 × 3 × 3.5 mm^3^). A high-resolution T1-weighted image was acquired for spatial normalization and visualization purposes (TR/TE 10/4.6 ms, flip angle 8°, matrix 304 × 299, FOV 24 cm, voxel size 0.75 × 0.75 × 0.8 mm^3^). Independent clinical neuroradiologists evaluated all T1 scans and no gross morphological or signal abnormalities were reported for any of the participants.

### fMRI pre-processing

fMRI data were preprocessed using the Statistical Parametric Mapping 8 (SPM8) software (Wellcome Dept. of Cognitive Neurology, http://www.fil.ion.ucl.ac.uk) running under the MATLAB R2012a programming and run-time environment (The Mathworks, Sherborn, MA, USA). First, functional images were realigned using rigid body transformations, followed by unwarping to remove residual distortions induced by movement and field inhomogeneity. None of the sessions contained images with a total linear displacement more than 3 mm in any direction. Average translation head motion was 1.05 mm, did not correlate with age (*r* = .047, *p* = .781) and was not significantly different between groups (*p* = .153). In addition, we calculated mean framewise displacement (FD) and the root mean square (RMS) of motion as reported by Power et al. (2012) and Van Dijk et al. (2012), respectively. Both were within acceptable limits [FD Power et al. = 0.210 (SD 0.097); RMS Van Dijk et al. = 0.048 (SD 0.022)] and did not differ between diagnostic groups (*p* = .210 and *p* = .241, respectively).

Next, slice-timing correction was performed to compensate for slice acquisition delays by temporally aligning all slices to the same reference time point (middle slice); given the interaction between timing shifts and motion, we chose performing realignment first to minimize the effect of inter-slice movement (Sladky et al. 2011). This step was followed by co-registration of the functional and structural images. T1-weighted images were segmented into grey and white matter. Then, functional and anatomical images were normalized to Montreal Neurological Institute (MNI) template (Friston et al. 1995). Finally, images were spatially smoothed with a Gaussian kernel of 6 mm at full width at half maximum.

### fMRI independent component analysis

Preprocessed time series were analyzed using the Group ICA of fMRI Toolbox (GIFT, http://icatb.sourcefourge.net, version 2.e) to identify spatially independent and temporally coherent networks (Calhoun et al. 2001, 2009). To minimize the impact of artifacts, we first ran ICA on each subject individually. After inspecting all images on the individual subject level, cleaned images of all 38 subjects were included in a Group ICA. The method is detailed in the following sections.

### Single subject analysis

Independent component (IC) estimation was performed using the Infomax algorithm (Bell and Sejnowski 1995), which was repeated 20 times in ICASSO in order to maximize the stability of the derived components (Himberg et al. 2004). The dimensionality of the data (number of networks) was estimated per subject using minimum description length (MDL) criteria tool built into GIFT. Images were back-reconstructed using GICA3, (Erhardt et al. 2011), which is a back-reconstruction method in which individual subject maps are reconstructed from the raw data using the ICA mixing matrix. Time series were then converted for visualization to reflect percent signal change. After single subject ICA, both the spatial pattern and the frequency spectrum of each component were inspected for the presence of possible image artifacts. Components containing obvious artifacts (e.g. edges, ventricles) were discarded.

### Group analysis

The cleaned data of all 38 subjects were carried forward to the group analysis. Group ICA was performed using the Infomax algorithm, which was repeated 20 times with ICASSO. All components showed high stability as indicated by the cluster quality index, *I*_q_ > 0.9. The number of components estimated through MDL was 44. Individual subject component maps were back-reconstructed using GICA3, and finally timecourses and spatial maps were normalized into *z*-scores (Beckmann et al. 2005).

### Selection of networks

We selected those components out of the initial 44 that reflected neuronal networks, based on the level of statistical significance and visual inspection for artifacts (McKeown et al. 1998; Calhoun et al. 2002b, 2004a, b; Kim et al. 2009; Meda et al. 2009a; Zhang and Li 2012). Five components were discarded as they showed a high spatial correlation with the probabilistic map of white matter or cerebrospinal fluid (*r* ^2^ > .025) provided in SPM8 while also showing low correlations with the cerebral grey matter map (*r* ^2^ < .05). Identification of the remaining components was performed through spatial multiple linear regression with established templates (Allen et al. 2011; Segall et al. 2012). Components with a spatial correlation greater than *r* ^2^ > .05 with template networks were carried forward to the final selection. Visual inspection of the 11 discarded components suggested that they represented eye movements, head motion or cardiac-induced pulsatile artifacts at the base of the brain.

To compute the degree of task-relatedness of the remaining 28 components, we regressed the corresponding timecourses against the design matrix (go and no-go stimuli together, along with their first temporal derivative) using the temporal multiple linear regression implemented in GIFT. The resulting beta weights (*β*) reflect the degree to which a component was modulated by the task events of interest. Beta weights of each IC for each task condition across the four runs were averaged per subject, and the group means of averaged *β* for each task condition were tested against zero using one-sample *t* tests (Zhang and Li 2012; Xu et al. 2013b). Eleven components were selected for the final analyses, with correlations significant at *p* < .001 with either go or no-go events. They were named according to the template they were spatially correlated with or based on visual inspection of the corresponding spatial map.

### Group differences in functional connectivity

Group differences in functional connectivity within the 11 selected components (intra-network connectivity) and among them (inter-network connectivity) were tested using the Mancovan toolbox (Allen et al. 2011) implemented in GIFT. We examined three connectivity measures: component spatial maps, component time course spectra, and between component functional network connectivity (Jafri et al. 2008). The voxel intensity in spatial map dictates the correspondence between a voxel time course and an IC time course (Balsters et al. 2013a); therefore, provides a measure of coactivation/synchronization (strength of connectivity) in a region within a given network. The spectra of time course reflect the degree of fluctuation in amplitude of the intrinsic activity captured by fMRI data within the network (Calhoun et al. 2012). Although ICs generated by ICA are maximally independent of each other (Calhoun and Adali 2006), their timecourses can still exhibit temporal dependencies (Arbabshirani et al. 2013): functional network connectivity evaluates the extent to which temporal coherence between networks is related to the variables of interest. A multivariate selection strategy was first performed in order to identify potential significant relationships between components measures and variables of interest: the initial design matrix included diagnosis and age as covariate, as well as an age-by-diagnosis interaction. In addition we included a head movement estimate as nuisance regressor (Allen et al. 2011; Balsters et al. 2013a, b), defined as the average of translation parameters, log-transformed for data normalization. Univariate analyses were performed within the reduced model to test for specific relationships between covariates of interest and connectivity properties. An alpha level of 0.05 was used for all analyses. Results were corrected for multiple comparisons using false discovery rate (FDR) (Genovese et al. 2002). Cohen’s *d* standardized effect sizes were calculated from corrected *p* values.

### Functional connectivity and clinical data correlations

In the sample of subjects with ASD, we analyzed the relationship between behavioral rigidity as measured by the RBS-R and functional connectivity measures (spatial maps, time course spectra, and functional network connectivity) of the 11 networks of interest selected for the group analysis. For this purpose, we ran a separate MANCOVA model with RBS-R total score and age as covariates, *p* = .05 FDR corrected for univariate testing.

## Results

### Task performance

All participants were able to successfully perform the task: mean accuracy on go trials was 99 % for both subjects with ASD and controls, and did not significantly differ between groups. Mean accuracy on no-go trials was 76 % (SD 0.15) for participants with ASD and 82 % (SD 0.12) for controls, did not differ between groups (*t* = 1.46, *p* = .153) and did not correlate with age. In line with findings from earlier studies using the same paradigm (Van Belle et al. submitted; Durston et al. 2002a), no-go accuracy decreased with the number of preceding go-trials (1, 3 or 5) for both children with ASD (83, 74, 72 %) and controls (87, 81, 78 %). Mean accuracy on no-go trials after 1, 3 or 5 go trials did not correlate with age and did not differ between ASD subjects and controls. Mean reaction time decreased with age (*r* = −.374, *p* = .022) and did not differ between groups (ASD 633 ± 104 ms, controls 620 ± 51 ms; mean ± SD). In the ASD group, RBS-R total score was not correlated with age or with any of the measures of task performance.

### Networks

**Fig. 1:**
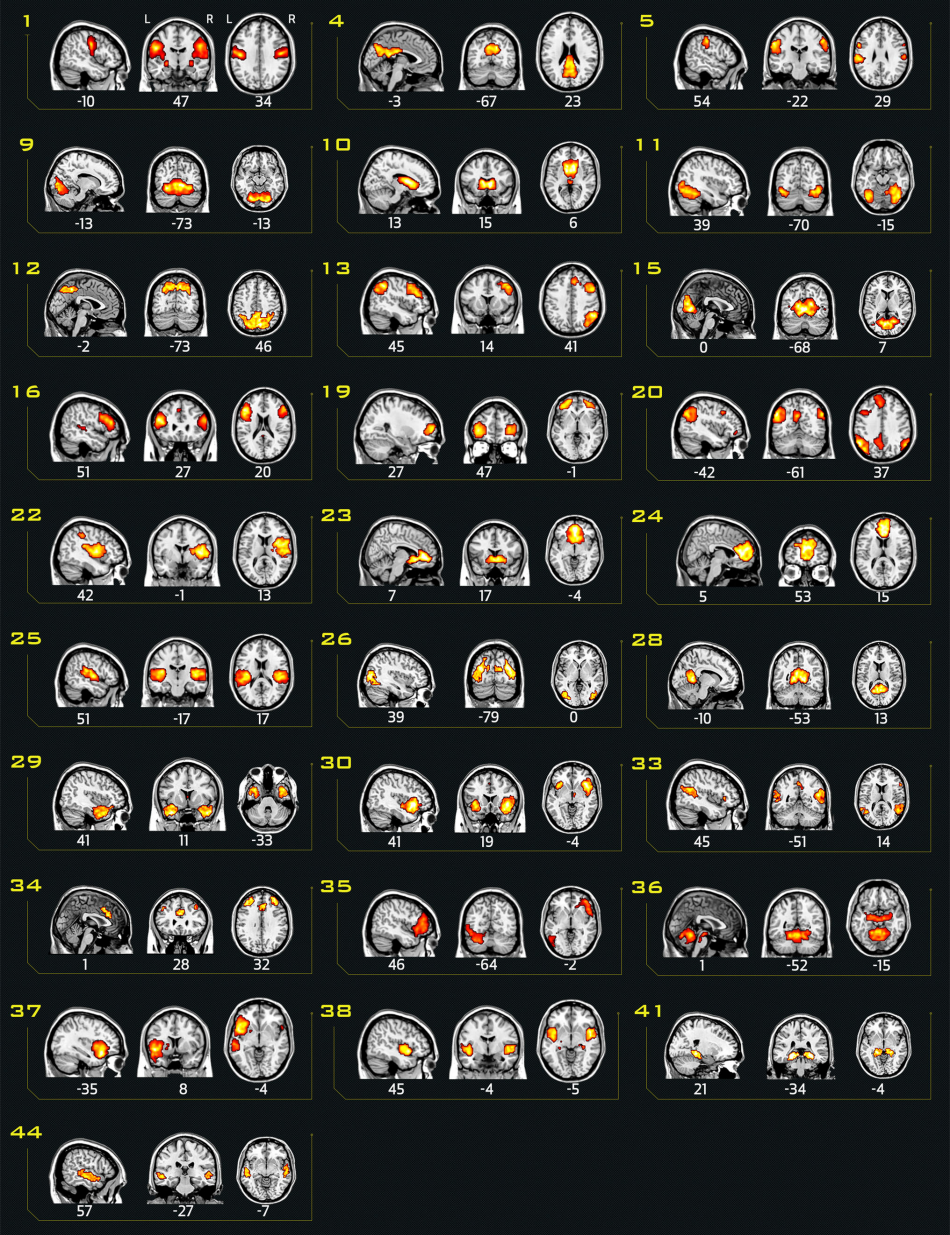
Overview of all independent components showing neural activity. The MNI coordinates refer to the slice intersections that are shown

**Fig. 2:**
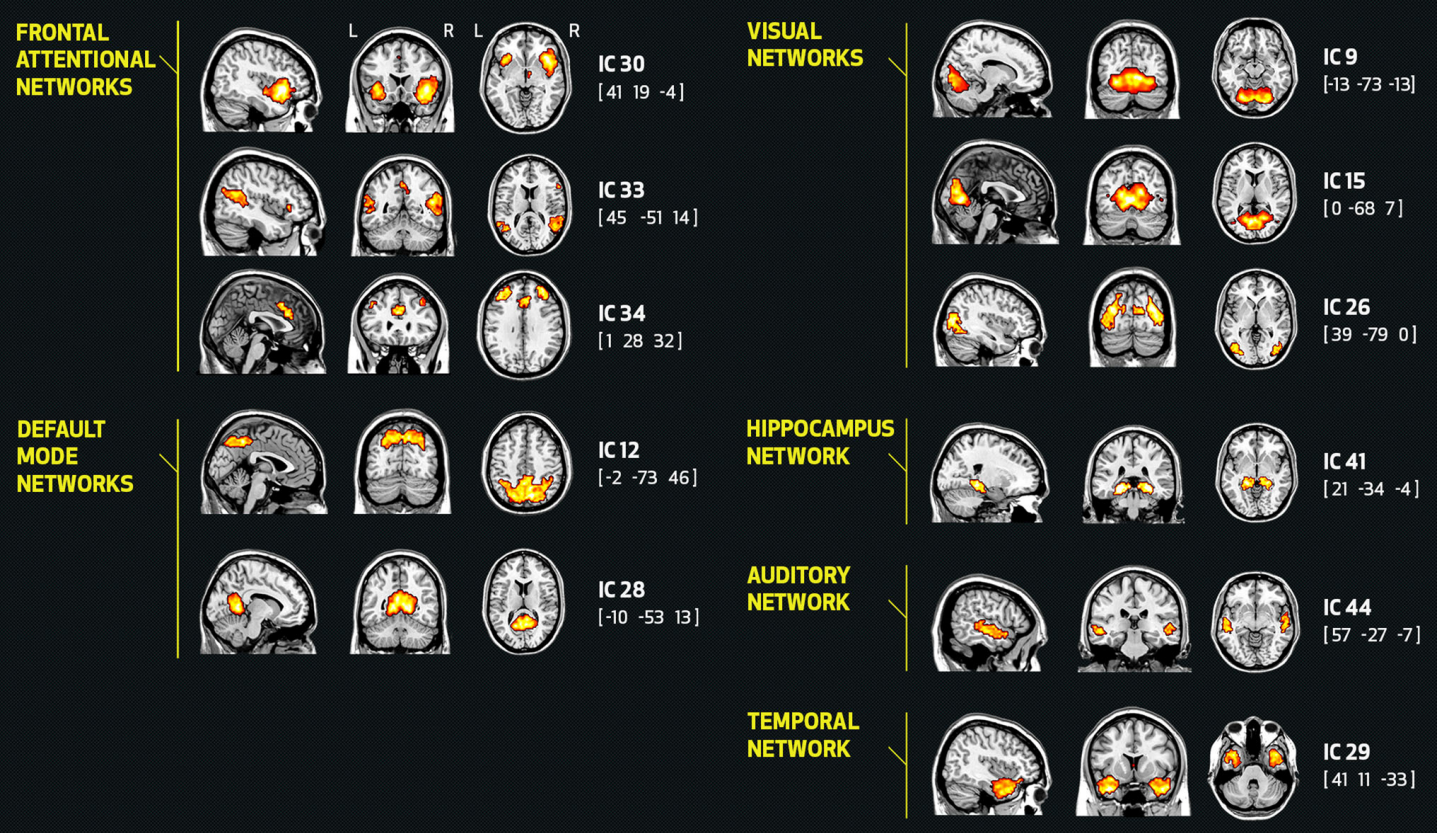
Networks of interest: frontal/attentional networks (ICs 30, 33, 34), default mode networks (ICs 12 and 28), visual networks (ICs 9, 15, 26), hippocampus network (IC 41), auditory network (IC 44) and temporal network (IC 29). The MNI coordinates refer to the slices shown, component labeling follows Allen conventions (Allen et al. 2011)

From the 28 IC containing neural networks (Fig. 1), 11 correlated with the task and were therefore identified as of interest for further analysis. These networks included frontal/attentional networks (IC 30, 33, 34), default mode networks (ICs 12 and 28), visual networks (ICs 9, 15, 26), a hippocampus network (IC 41), an auditory network (IC 44) and a temporal network (IC 29) (Fig. 2).

We assessed *β*-values to ascertain the degree of engagement of networks during go or no-go events (Meda et al. 2009b). The analyses showed that activity in IC 30 and IC 34 (frontal/attentional network) were related to no-go events, while the default mode network components were anti-correlated with both go and no-go events (Online Resource 1).

### Functional connectivity

**Fig. 3:**
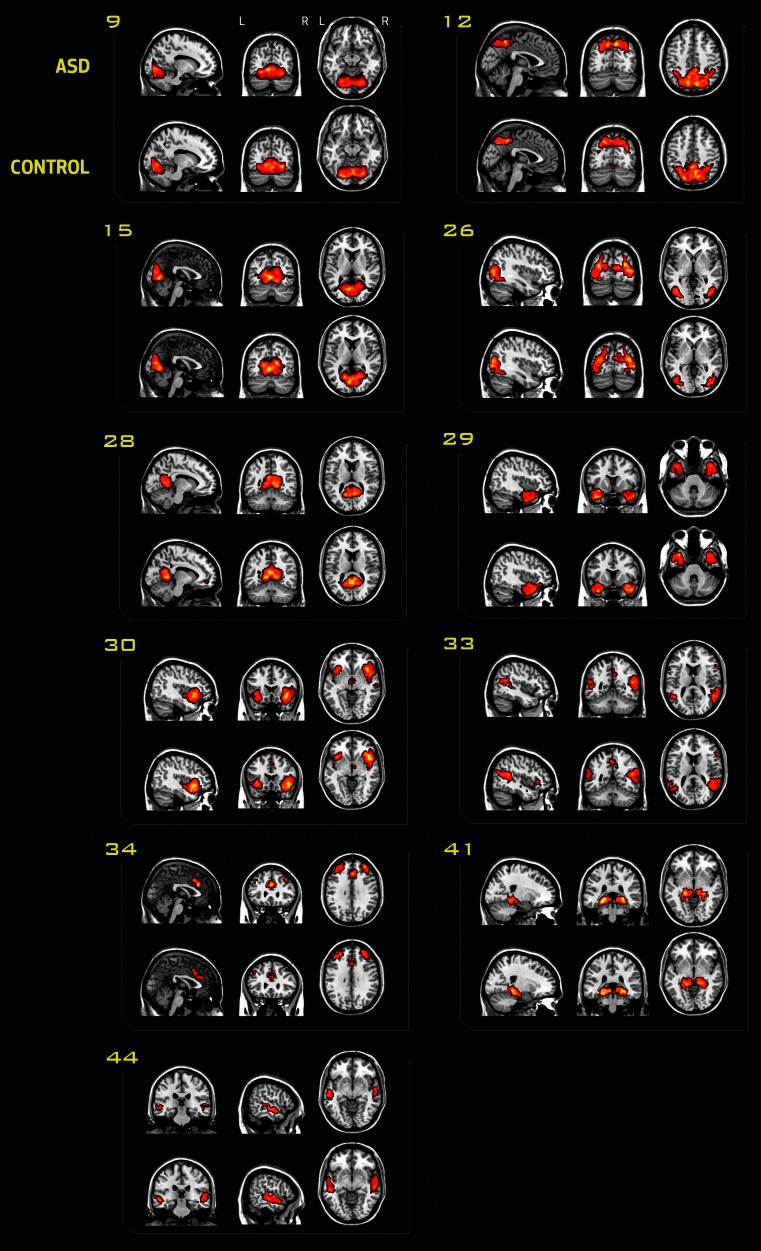
Networks of interest in subjects with ASD and typically developing controls. Component spatial maps of the networks of interest are shown in both groups separately to illustrate the between-group similarities. For each network, the first row of images belongs to the ASD group and the second row to the control group. The MNI coordinates refer to the slices shown

Multivariate and univariate tests showed no effect of diagnosis on the spatial map of components, the timecourse spectra or between-network connectivity, with only small effect sizes (ranging from *d* = 0.17 to 0.23). As there was no significant main effect of diagnosis or age, we reran the MANCOVAN analysis without the interaction term. The results remained non-significant. Spatial maps of the networks of interest are depicted in Fig. 3, illustrating the similarities between groups.

We ran a power analysis to estimate the sample size that would be needed to show between-group differences if there was in fact a meaningful difference (pFDR <0.05, 2-sided). This told us that a sample of *N* = 788 would be required to reach a power level of 0.80 and confirmed our conclusion that any differences were minimal and more likely related to noise.

In addition, we performed a standard GLM analysis of the fMRI task, which further confirmed that there were no differences between groups in brain activation during performance of the task (details are provided in Online Resource 2).

**Fig. 4:**
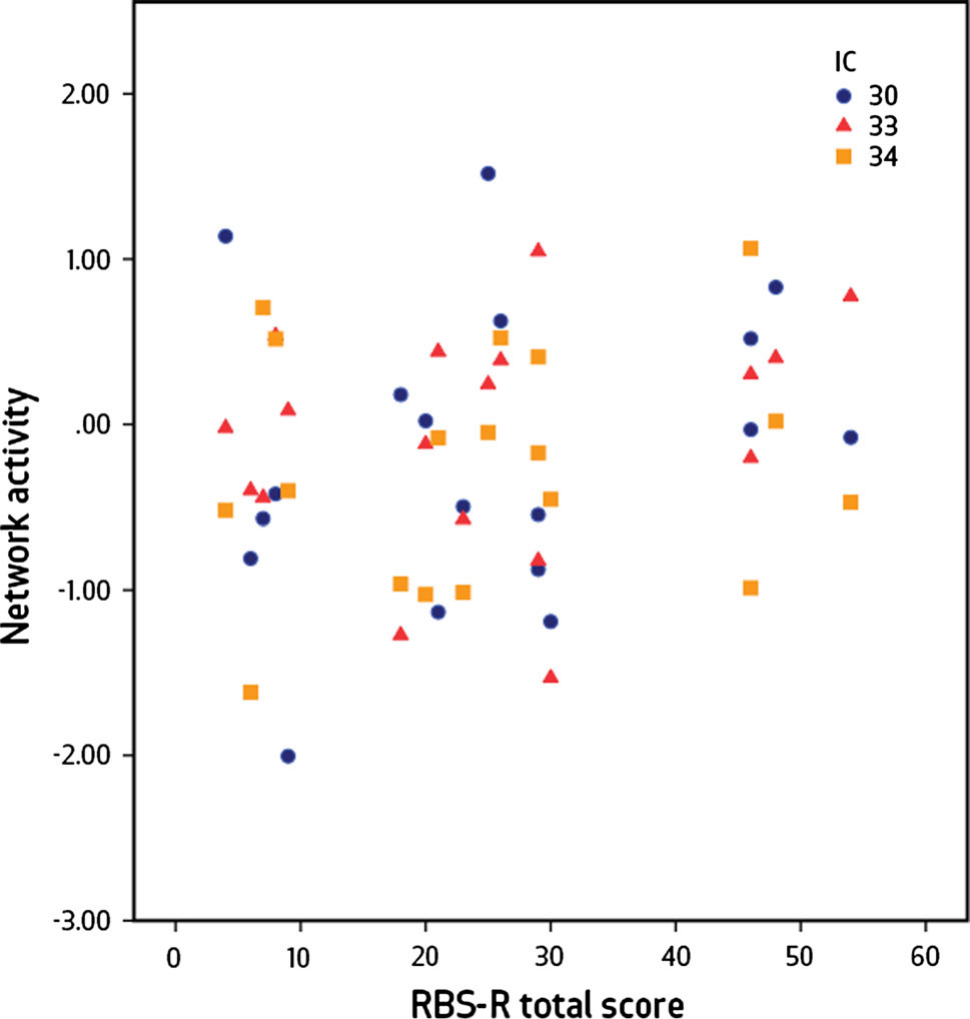
There is no relation between total RBS-R score and activity in frontal/attentional networks

We found no significant correlation between the RBS-R total score of subjects with ASD and functional connectivity measures of the components of interest. Average activity from the networks of interest was plotted against total RBS-R scores to illustrate the lack of relation (Fig. 4).

## Discussion

In this study, we examined functional connectivity during the performance of a cognitive control task (go/no-go) in a population of high-functioning boys with ASD and age-matched typically developing boys using a multivariate data-driven approach (ICA). We found no evidence for changes in functional connectivity in ASD. This is consistent with ROI-based research in children performing a similar paradigm (Lee et al. 2009), but contrasts with studies of adolescents and adults with ASD that have reported decreased functional connectivity in cognitive control networks (Just et al. 2007; Kana et al. 2007; Agam et al. 2010; Solomon et al. 2009).

The findings of dysfunctional connectivity in adults, but not in children, with ASD suggests that changes in connectivity patterns related to cognitive control may appear relatively late in the disorder. This is in keeping with research showing that immature brain activity may be characterized by less structured and more diffuse patterns than in adults (Durston et al. 2006; Supekar et al. 2009). It also implies that detecting subtle differences in the functional connectivity of cognitive control network between children with ASD and typically developing controls may be a particularly difficult challenge. Perhaps it is therefore not entirely surprising that the present study converges with an increasing body of literature reporting only limited changes in functional connectivity in children with ASD, both during rest (Bos et al. under revision) and cognitive control (Lee et al. 2009). Furthermore, we found no evidence for an association between the severity of rigid behavior in our subjects and either functional connectivity or task performance.

There are some strong points to our study, but also some limitations that need to be taken into consideration. One strong point is that we standardized our data analysis as much as possible to limit the number of arbitrary decisions. We did this by using a data-driven approach (ICA) and a hypothesis-free procedure for network selection. A weak point is the limited sensitivity of the RBS-R questionnaire to detect symptoms of rigidity in typically developing subjects. Therefore, correlations between rigidity and functional connectivity measures could only be assessed in children with ASD. A possible further limitation was our relatively small sample size. However, the two groups were well matched and similar to samples in other reports on functional connectivity (Just et al. 2007; Kana et al. 2007; Agam et al. 2010; Solomon et al. 2009). Furthermore, a post hoc power analysis showed that we would need an enormous number of subjects (788) to show between-group differences in connectivity on this task if there is indeed a true difference. This further supports our interpretation that differences in functional connectivity of cognitive control networks between typically developing children and children with ASD are minimal. In conclusion, we assessed functional connectivity in a well-characterized cohort of children with and without ASD during the performance of a cognitive control task, using a data-driven multivariate approach. We confirmed previous findings of no differences in connectivity in children with ASD. These findings do not support hypotheses that there are changes in cognitive control and the networks underlying it in children with ASD.

## Electronic supplementary material

Below is the link to the electronic supplementary material.

Supplementary material 1 (PDF 52 kb)

Supplementary material 2 (PDF 288 kb)
