# Supplementary material for: Functional connectivity during cognitive control in children with autism spectrum disorder: an independent component analysis
Source: J Neural Transm (Vienna). 2014 May 21;121(9):1145–55. doi: 10.1007/s00702-014-1237-8 (PMC4141973; doi:10.1007/s00702-014-1237-8)
Supplement: Supplementary file 1 — Supplementary material 1 (PDF 52 kb) [file 702_2014_1237_MOESM1_ESM.pdf]

Journal of Neural Transmission

Functional connectivity during cognitive control in children with autism spectrum disorder: an independent component analysis

S. Ambrosino, D.J. Bos, T.R. van Raalten, N.A. Kobussen, J. van Belle, B. Oranje, S. Durston

Corresponding author**:**
Sara Ambrosino, NICHE Lab, Department of Psychiatry, Brain Centre Rudolf Magnus, University Medical Centre Utrecht, The Netherlands; E-mail: S.AmbrosinodiBruttopilo-3@umcutrecht.nl

Electronic Supplementary Material 1

Relationship between activity in networks and task events (go and no-go trials)

**Components**: name	number	β: M/SD		p-value correlation with task[a]	
		**go**	**no-go**	**go**	**no-go**
**Frontal / Attentional networks**	**30**	.71/2.31	2.55/2.61	.065	<.001
	**33**	-2.04/2.79	-1.47/2.76	<.001	.002
	**34**	.44/2.94	1.93/3.12	.364	<.001
**Default mode networks**	**12**	-2.89/3.13	-2.76/3.33	<.001	<.001
	**28**	-2.48/2.53	-3.72/2.74	<.001	<.001
**Visual networks**	**9**	-2.66/3.26	-3.49/3.14	<.001	<.001
	**15**	-5.07/3.40	-4.87/3.20	<.001	<.001
	**26**	-2.23/2.41	-3.37/2.50	<.001	<.001
**Hippocampus network**	**41**	-1.12/1.57	-1.09/1.83	<.001	.001
**Auditory network**	**44**	-.69/2.19	-1.40/2.29	.058	<.001
**Temporal network**	**29**	-1.61/1.91	-2.11/2.16	<.001	<.001

[a]One-sample t-test to determine whether β differed from zero, uncorrected p-values
