# Supplementary material for: Functional connectivity during cognitive control in children with autism spectrum disorder: an independent component analysis
Source: J Neural Transm (Vienna). 2014 May 21;121(9):1145–55. doi: 10.1007/s00702-014-1237-8 (PMC4141973; doi:10.1007/s00702-014-1237-8)
Supplement: Supplementary file 2 — Supplementary material 2 (PDF 288 kb) [file 702_2014_1237_MOESM2_ESM.pdf]

Journal of Neural Transmission

Functional connectivity during cognitive control in children with autism spectrum disorder: an independent component analysis

S. Ambrosino, D.J. Bos, T.R. van Raalten, N.A. Kobussen, J. van Belle, B. Oranje, S. Durston

Corresponding author:
Sara Ambrosino, NICHE Lab, Department of Psychiatry, Brain Centre Rudolf Magnus, University Medical Centre Utrecht, The Netherlands; E-mail: S.AmbrosinodiBruttopilo-3@umcutrecht.nl

Electronic Supplementary Material 2

GLM analysis of fMRI data during task performance

Methods

As described in the main paper, the fMRI data were preprocessed using a standard pipeline in SPM8 (Wellcome Dept. of Cognitive Neurology, www.fil.ion.ucl.ac.uk). Following this, the preprocessed data were cleaned before the statistical analyses using single-subject ICA. This is an effective method for removing noise from the data, and as such is also advantageous to GLM analyses.

fMRI task analyses - GLM

The design matrices used in the GLM analyses were identical to the ones we used in the ICA analysis (see the main paper for a description). At the first level, five event types were defined: initial fixation, go trials, correct and incorrect no-go trials, and a parametric factor representing the number of go trials preceding a no-go event. These events included two events of interest (go and no-go trials) and three events of no interest (errors, fixation and the parametric factor). The event-types were time-locked to stimuli by a canonical synthetic hemodynamic response function (HRF) and its first-order temporal derivative (tHRF).

For the group analysis, a random effects model was used in SPM8 to compute a voxelwise T-statistic for the contrast no-go trials > go trials. Regions of Interest included the all

voxels that were activated in the ASD and typically developing group during the no-go > go condition, tested with a one-sample T-test with age as covariate, at a threshold of $p < .001$ (uncorrected), $k > 10$ voxels. Group differences in activation were again tested with a random effects model, using two-sample T-tests at a False Discovery Rate-corrected threshold of $p < .05$, with age entered as a covariate to the design.

Results

fMRI task analyses – GLM results

The one-sample T-test over all participants showed the usual pattern of activation in cognitive control areas, such as the anterior cingulate cortex, bilateral inferior frontal gyrus, bilateral orbitofrontal gyrus, bilateral insula and areas in the bilateral inferior parietal cortex (Supplemental Fig. 1). Two-sample T-test showed that there were no group differences in activation during no-go trials, even when the threshold was liberalized to $p < .001$ (uncorrected), $k > 10$ voxels. Taken together, these results indicate that our paradigm successfully engaged cognitive control areas, both for children with ASD and typically developing controls and that there were no differences in brain activity between diagnostic groups.

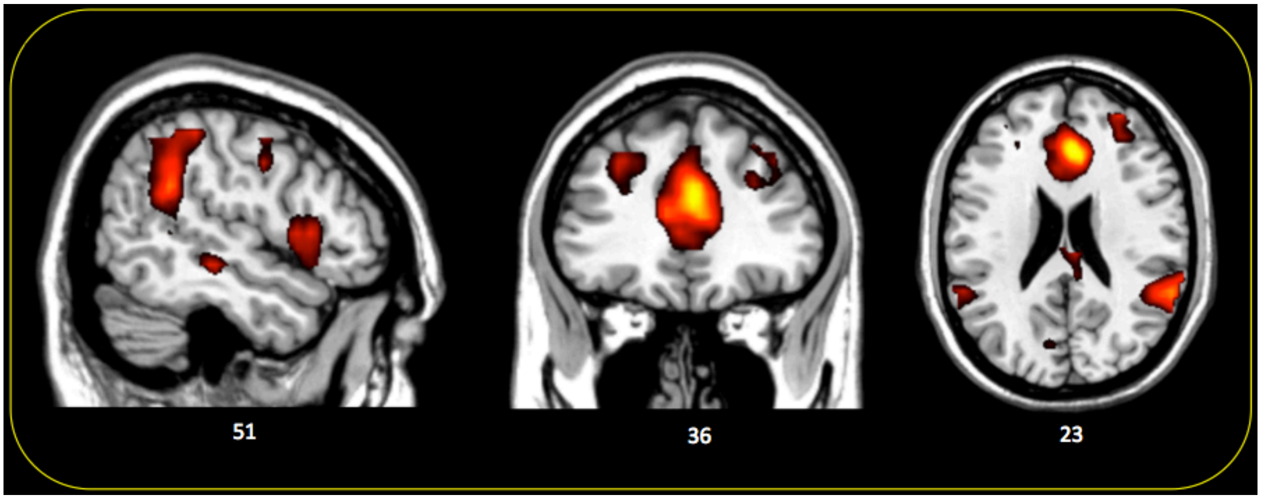

Supplemental Fig. 1 Activation in cognitive control areas during no-go trials. Maps of brain activity for the whole group for the contrast no-go > go, SPM(T) overlaid on a single subject T1 template. The MNI slice location is provided below each image.
